# Efficacy of botulinum toxin type B (rimabotulinumtoxinB) in patients with cervical dystonia previously treated with botulinum toxin type A: A post-marketing observational study in Japan

**DOI:** 10.1016/j.ensci.2021.100374

**Published:** 2021-10-27

**Authors:** Ryuji Kaji, Akira Endo, Michiko Sugawara, Mika Ishii

**Affiliations:** a National Hospital Organization Utano National Hospital, 8 Narutaki Ondoyama-cho, Ukyo-ku, Kyoto, Japan; b Institute of Health Biosciences, Tokushima University Graduate School, 3-18-15 Kuramotocho, Tokushima, Japan; c Clinical Planning and Development, Medical HQs, Eisai Co., Ltd., 4-6-10 Koishikawa, Bunkyo-ku, Tokyo, Japan; d Scientific Intelligence Group, Medical HQs, Eisai Co., Ltd., 4-6-10 Koishikawa, Bunkyo-ku, Tokyo, Japan

**Keywords:** Cervical dystonia, Post-marketing observational study, Botulinum toxin type A, Botulinum toxin type B, Toronto Western Spasmodic Torticollis Rating Scale, AE, adverse effects, CD, cervical dystonia, CI, confidence interval, MedDRA, Medical Dictionary for Regulatory Activities, TWSTRS, Toronto Western Spasmodic Torticollis Rating Scale

## Abstract

To date, efficacy data on botulinum toxin type B (rimabotulinumtoxinB) in patients with cervical dystonia (CD) previously treated with botulinum toxin type A in a large population are lacking; thus, we aimed to evaluate type B efficacy in this patient population. In a post-marketing observational cohort study, 150 patients previously treated with botulinum toxin type A were enrolled, of whom 138 were followed up for 1 year after the initial type B injection. Final observation data were available for 122 patients. Efficacy was evaluated using the Toronto Western Spasmodic Torticollis Rating Scale. Total score improved from 39.9 at baseline to 34.3 at 4 weeks after the first injection, and pain score improved from 8.9 to 7.9. Improvements were maintained through six further injections in two subpopulations: patients who showed resistance to botulinum toxin type A and patients who were not type A resistant but switched to type B. For a number of patients, even low doses (<5000 units) of botulinum toxin type B demonstrated efficacy. These findings support the efficacy of botulinum toxin type B in clinical settings for the management of CD symptoms, including pain, even at low doses, regardless of the patient's botulinum toxin type A resistance status.

## Highlights

• Botulinum toxin type B improved cervical dystonia symptoms and pain after 4 weeks.
• Botulinum toxin type B was effective even at low doses (<5000 units).
• The efficacy of toxin type B is not affected by toxin type A resistance status.

## 1 Introduction

Cervical dystonia (CD) is a chronic movement disorder characterized by sustained involuntary muscle contractions leading to pain, abnormal head and neck positioning, functional impairment, and compromised quality of life [1,2]. The primary treatment of CD is injection with botulinum toxin into the dystonic muscles. Among the serotypes of botulinum toxin available for therapeutic purposes, type A is typically used first while type B (rimabotulinumtoxinB) can be used in patients having an inadequate response to type A; type B, therefore, represents an alternative option for patients with type A resistance. In the Japanese CD treatment guidelines, type A and type B are similarly recommended for CD [3,4]. Both types have been evaluated for efficacy and safety and are recommended in international clinical guidelines for the treatment of CD [[3], [4], [5], [6], [7], [8], [9], [10]].

Previous studies have compared the efficacy and safety of botulinum toxin type B with botulinum toxin type A for the treatment of CD [8,11,12]. However, there is insufficient clinical evidence to support the preferential use of one form of botulinum toxin over the other [8]. Both serotypes were observed to have a similar duration of efficacy, and there was no significant difference between the groups with respect to Toronto Western Spasmodic Torticollis Rating Scale (TWSTRS) score, a composite scale used to measure severity, disability, and pain in CD [13]; however, dry mouth and swallowing difficulties were more prevalent in the type B group. As previously mentioned, the Japanese guidelines recommend both types of botulinum toxin for the treatment of CD [3,4]. Globally, treatment with type B is a therapeutic option for patients who develop resistance to botulinum type A with repeated use, and the safety and efficacy of this approach compared with placebo has been confirmed in a study of type A-resistant patients with CD [14]. In Japan, however, type B has been evaluated in very few type A-resistant patients to date [15]. It is therefore imperative to determine the real-world efficacy of type B in patients previously treated with botulinum toxin type A in clinical settings.

Here, we report the results of a post-marketing observational study in Japan in patients with CD who switched treatment from botulinum toxin type A to type B. The study was performed to meet regulatory obligations that require manufacturers of pharmaceutical agents to conduct post-marketing studies to accumulate safety and efficacy data on the agent in routine clinical practice [16]. The purpose of this study was to evaluate the efficacy of type B in this patient population using TWSTRS score at 4-week intervals after each injection for a period of 1 year. Furthermore, we examined the effect of type B dose and presence or absence of resistance to prior treatment with botulinum toxin type A to clarify the optimal use of type B in clinical settings.

## 2 Methods

### 2.1 Study design and patients

This was a multi-center, post-marketing, observational study conducted at hospital neurology and neurosurgery centers in Japan. The study is registered at ClinicalTrials.gov with the identifier NCT02175719. Patients who had previously been treated with botulinum toxin type A for CD were eligible for inclusion. Patients with botulinum toxin type A-resistant CD as well as those who were not type-A resistant but were switched to type B according to the physician's decision were enrolled. Exclusion criteria included the absence of TWSTRS assessment immediately prior to the first dose of type B and a period of <2 months between the last dose of botulinum toxin type A and the first dose of type B. The study registration period was from 27 March 2013 to 30 November 2016 and the study period was from 27 March 2013 to 31 December 2017. Data were collected using case report forms and centralized information documentation. This study was conducted in compliance with Japanese Good Post-Marketing Study Practice. While patient informed consent was not required for this post-marketing study in accordance with Japanese regulations, patients were informed of the treatment objectives and that they would be receiving treatment with botulinum toxin type B. Patients were given the opportunity to refuse consent to this treatment. Written informed consent to receive botulinum toxin type B treatment was obtained.

### 2.2 Intervention

Participants received an injection of type B into the affected muscles at an initial dose up to 5000 units. In the case of multiple tonic muscles, the drug was injected into each muscle in divided doses. Dosing was in accordance with the prescribing information. In patients who did not experience sufficient efficacy or reported recurrent symptoms, up to 10,000 units in total were permitted in subsequent injections. However, re-injection within 2 months of the initial dose was discouraged. Target muscles included the sternocleidomastoid, scalenus, trapezius, levator scapularis, splenius capitis, and semispinalis capitis. The recommended initial and maximum dose of type B for each target muscle type is shown in Table S1. The precautions for dosage and administration can be found as Supplementary information. The guidance for type B injection was not specified and depended on the physician's decision.

### 2.3 Efficacy evaluation

The efficacy of type B for CD was evaluated using TWSTRS [17]. The TWSTRS pain, disability, and severity subscores and total score were calculated on the day prior to the type B injection and every 4 weeks thereafter for a period of 1 year from the first injection.

Subgroup analyses were performed for the maximum dose of type B during the study period, stratified by lower (≤5000 units) and higher (>5000–10,000 units) maximum dose. Furthermore, subgroup analysis stratified by presence or absence of botulinum toxin type A resistance was conducted.

Type B was not specifically assessed for safety in the present study; safety data are instead being evaluated in another post-marketing study (NCT02175693) that includes all patients from the present study. However, the data collected for adverse reactions are presented herein. The reasons for discontinuation, including adverse effects (AEs), were recorded. AEs were classified according to the Medical Dictionary for Regulatory Activities (MedDRA/J), ver.21.0.

### 2.4 Statistical analysis

The planned sample size was 150 patients, based on the feasibility of enrolling patients with the requisite TWSTRS data from a limited number of institutions, such that there would be at least 100 efficacy evaluable patients; as this study was not designed to test a hypothesis, no detailed power calculations were necessary. Summary statistics were calculated for the TWSTRS score at baseline (immediately prior to administration of the first dose of type B), and before and after injection at each 4-week interval after subsequent administrations. The change in score from baseline and corresponding 95% confidence interval (CI) was calculated for each timepoint. The final observation was defined as the evaluation at 4 weeks after the last injection.

A visit window of ±2 weeks was permitted for the evaluation at Week 4 to reflect real-world clinical practice. The observation period was defined as 1 year, but evaluations that took place later than that were included if they were captured in the case report form.

## 3 Results

### 3.1 Patient characteristics

**Supplementary Fig. S1:**
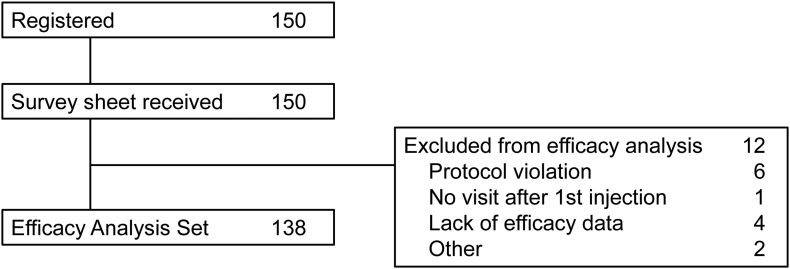
Flow chart of patient inclusion in the efficacy analysis set.

A total of 150 patients were enrolled from 17 hospitals, and 138 of these patients were included in the efficacy analysis set (Fig. S1). The baseline characteristics of the 138 included patients are shown in Table 1. The mean ± standard deviation age of participants was 55.6 ± 13.1 years, and 71 (51.45%) were male. Regarding the efficacy of prior botulinum toxin A use, half of patients (*n* = 69, 50.00%) were classified as resistant; this included 37 (26.81%) with a “diminished effect” (defined as effective at the first injection, but ineffective at the second or later injection) and 32 (23.19%) with an “ineffective at the 1st injection”. A further 63 (45.65%) patients had an “effective” classification, and data were lacking for the remaining six (4.35%) patients.

In the efficacy analysis set, 38.41% (*n* = 53) of patients discontinued treatment for the following reasons: ineffective at the first type B injection (*n* = 26), lost to follow up (*n* = 13), or AEs (*n* = 8). Of 26 patients for whom type B was ineffective at the first dose, 10 were refractory to type A and type B, including 5 of 13 patients who discontinued at the first dose. However, few patients discontinued due to a diminished effect subsequent to the first injection of type B (*n* = 3, Table 2). Of the 69 patients who were classified as resistant to botulinum toxin A at baseline, 52 had data available for analysis at Week 4.

**Table 1:** Baseline characteristics of patients.

Characteristics	*n* (%)
Efficacy analysis set	138

Sex	
Male	71 (51.45)
Female	67 (48.55)

Age, years	
<65	101 (73.19)
≥65	37 (26.81)
Mean ± SD	55.6 ± 13.1
Median (min, max)	56.5 (28, 85)

Disease duration	
<1 year	4 (2.9)
1 to <3 years	14 (10.14)
3 to <5 years	21 (15.22)
5 to <10 years	35 (25.36)
≥10 years	62 (44.93)
Unknown	2 (1.45)

Response to prior botulinum toxin type A treatment	
Effective	63 (45.65)
Diminished effect^a^	37 (26.81)
Ineffective at the 1st injection	32 (23.19)
Unknown	6 (4.35)

Botulinum toxin type A treatment period, days	
Patients, *n*^b^	76
Mean ± SD	1308.1 ± 1362.4
Median (min, max)	813 (1, 4858)

Botulinum toxin type A antibody	
Absence	0
Presence	2 (1.45)
Unknown	136 (98.55)

SD, standard deviation.

a Defined as effective at the first injection but ineffective at the second or later injection.

b Excludes patients whose dosing period was unknown.

**Table 2:** Patient discontinuation.

	*n* (%)
Efficacy analysis set	138

Discontinued	
No	85 (61.59)
Yes	53 (38.41)

Reason	
No clinic visit	13 (9.42)
Adverse event	8 (5.8)
Diminished effect	3 (2.17)
Ineffective at the 1st injection	26 (18.84)
Other	6 (4.35)

### 3.2 Injection number and type B dosage

The mean number of type B injections was 3.6 ± 1.8; 79 patients (57.25%) received four or more injections and 31 patients (22.46%) received six injections (Table 3). The mean maximum dose was 5746.0 ± 2475.9 units. The mean number of injections was 3.6 ± 1.8 (median: 4.0 [range: 1–6]).

**Table 3:** Dosage and injection interval.

Number of injections		1st	2nd	3rd	4th	5th	6th
Number of patients		138	112	90	79	48	31
Dosage (units)	Mean ± SD	3827.9 ± 1409.0	5375.4 ± 2297.3	5870.6 ± 2511.6	5916.9 ± 2601.0	5875.0 ± 2373.4	5887.1 ± 2096.5
	Median (min, max)	4875.0 (1000, 10,000)	5000.0 (2400, 10,000)	5000.0 (2500, 10,000)	5000.0 (2500, 10,000)	5000.0 (2500, 10,000)	5000.0 (2500, 10,000)
Injection interval (days)	Mean ± SD	–	97.1 ± 34.7	92.4 ± 33.7	90.6 ± 23.0	83.9 ± 18.5	84.4 ± 17.5
	Median (min, max)	–	91.0 (60, 202)	91.0 (62, 252)	91.0 (62, 196)	84.0 (62, 133)	84.0 (62, 119)

SD, standard deviation.

The dosages and injection intervals for the 138 patients in the efficacy analysis set are shown in Table 3. The median first dose was 4875.0 units and the median second and subsequent doses were 5000.0 units. The median interval was 91.0 days for the second to the fourth injections, and 84.0 days for the fifth and sixth injections.

### 3.3 Change in TWSTRS total score and pain, severity, and disability subscores

**Fig. 1:**
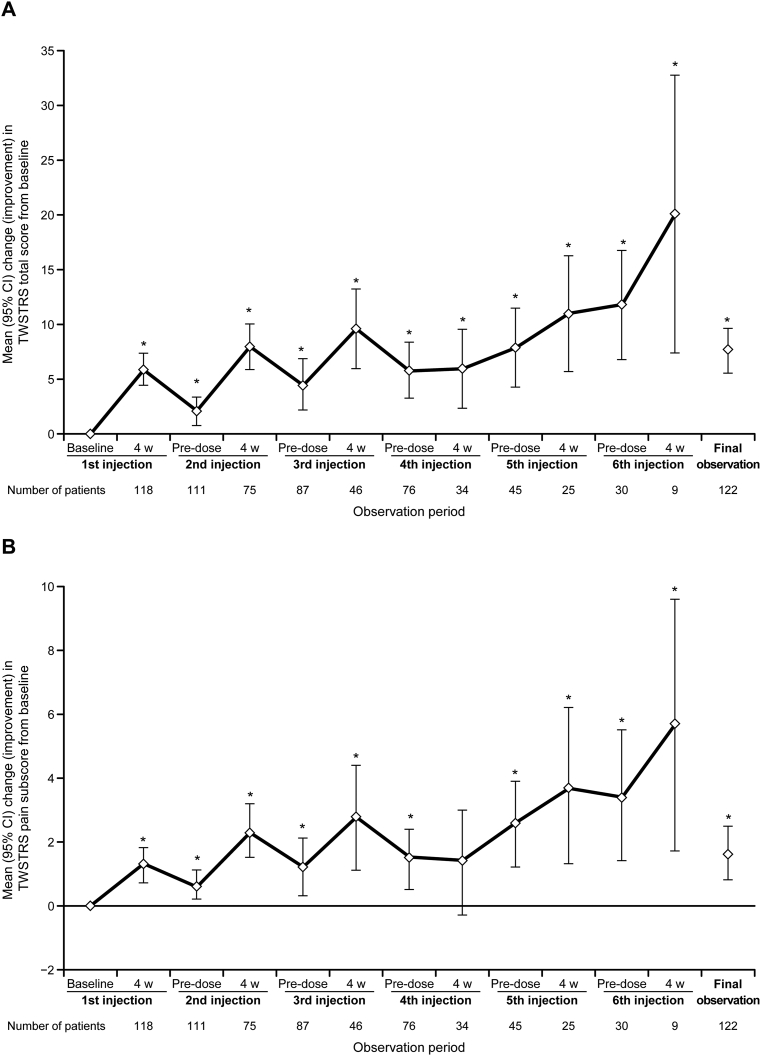

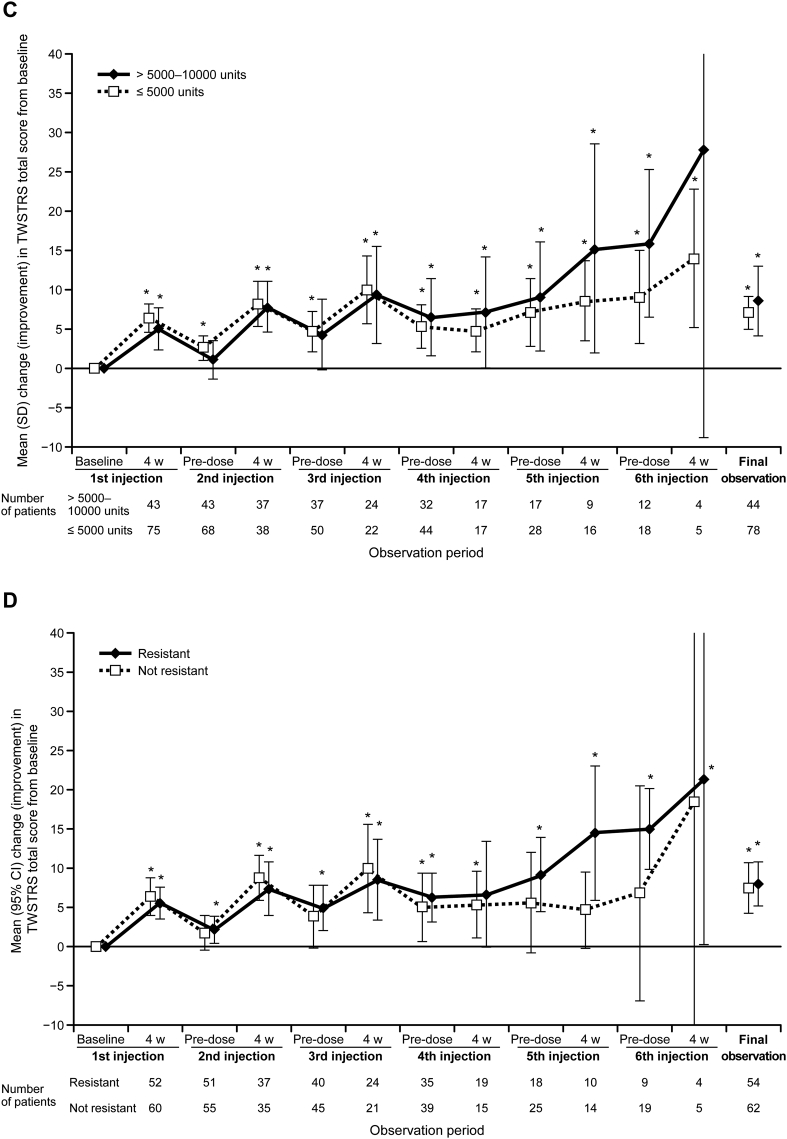
Mean (95% CI) change (decrease) from baseline in Toronto Western Spasmodic Torticollis Rating Scale (TWSTRS) total score for patients in the efficacy analysis set (a), TWSTRS pain subscore for patients in the efficacy analysis set (b), TWSTRS total score by dose subgroup (c), and TWSTRS total score by botulinum toxin type A-resistance status (d). The final observation was 4 weeks after the last injection. *p < 0.05. CI, confidence interval; w, weeks.

Changes (decreases) in TWSTRS total score reflecting the improvement of symptoms are shown in Fig. 1A. No data were available at Week 4 for 20 of the 138 patients in the efficacy analysis set because the evaluation was not performed (*n* = 7) or took place more than 6 weeks after injection (*n* = 11), or the patient received an initial dose >5000 units (*n* = 2). TWSTRS scores were therefore available for 118 patients at Week 4 after the first injection, and the mean TWSTRS total score improved from 39.9 at baseline to 34.3 at Week 4. Similarly, the TWSTRS total score showed a marked improvement at 4 weeks after the second and third injections compared with the baseline score. The mean decrease in TWSTRS total score from baseline to 4 weeks after the first to sixth injection was 5.9 (95% CI: 4.5–7.4), 8.0 (95% CI: 5.9–10.1), 9.7 (95% CI: 6.0–13.3), 6.0 (95% CI: 2.4–9.6), 11.0 (95% CI: 5.7–16.3), and 20.1 (95% CI: 7.4–32.8), respectively, and the improvement in total score was maintained until the sixth injection. Final observation data were collected for 122 patients, and the mean decrease in TWSTRS total score from baseline in these patients was 7.7 (95% CI: 5.6–9.7).

The mean TWSTRS pain subscore also decreased from 8.9 (baseline) to 7.9 (Week 4), and a clear improvement was subsequently observed after the second and third injections (Fig. 1B). The mean TWSTRS pain subscore tended to decrease until the sixth injection, and the mean TWSTRS pain subscore at the final observation was decreased by 1.6 (95% CI: 0.8–2.5) from baseline.

**Supplementary Fig. S2:**
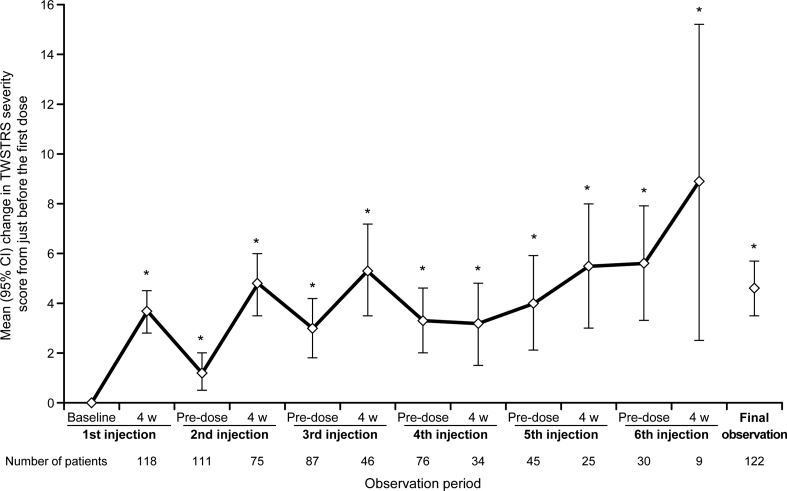
Mean (95% CI) change (decrease) in TWSTRS severity score from baseline. The final observation was 4 weeks after the final injection. *p < 0.05. CI, confidence interval; TWSTRS, Toronto Western Spasmodic Torticollis Rating Scale; w, weeks.

**Supplementary Fig. S3:**
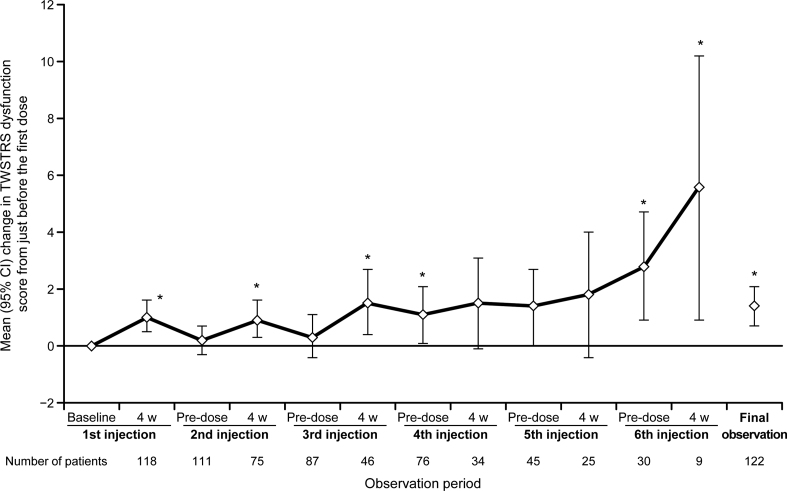
Mean (95% CI) change (decrease) in TWSTRS dysfunction score from baseline. The final observation was 4 weeks after the final injection. *p < 0.05. CI, confidence interval; TWSTRS, Toronto Western Spasmodic Torticollis Rating Scale; w, weeks.

Mean TWSTRS severity subscore also decreased from 18.9 (baseline) to 15.3 (Week 4) (Fig. S2), while mean TWSTRS dysfunction subscore decreased from 12.1 (baseline) to 11.1 (Week 4) (Fig. S3). Both scores tend to decrease until the sixth injection, indicating consistent improvements with long-term treatment.

### 3.4 Subgroup analysis by type B dose and type A-resistance status

The subgroup analysis of mean TWSTRS total score stratified by doses of ≤5000 units and >5000–10,000 units is shown in Fig. 1C. The mean TWSTRS total score at baseline for the lower maximum dose (≤5000 units) and the higher maximum dose (>5000–10,000 units) was 38.2 and 43.5 and decreased 4 weeks after the first injection to 31.6 and 38.8, respectively. Similarly, after the second and third injections, TWSTRS total score showed an improvement in both dose groups from baseline, and the tendency for this improvement was maintained up to the sixth injection.

Changes in TWSTRS total score for patient subpopulations with or without resistance to botulinum toxin type A are shown in Fig. 1D. A similar improvement from baseline in total score after each injection was observed in both subpopulations, and this improvement tended to be maintained until the sixth injection in both subpopulations. These results indicate that the efficacy of type B was not affected by prior botulinum toxin type A resistance.

### 3.5 Safety

A summary of adverse reactions is provided in Table 4. The most frequently reported adverse reactions were thirst (*n* = 6 [4.35%]), injection site pain (*n* = 3 [2.17%]), dysphagia (*n* = 2 [1.45%]), and neck pain (*n* = 2 [1.45%]). The main reason for discontinuation was thirst. Eight patients discontinued treatment as a result of one or more AEs, which included thirst (*n* = 4), neck pain (*n* = 2), and rash, injection site pain, vision blurred, and malaise (*n* = 1 each). No serious AEs were reported in this study.

**Table 4:** Adverse reactions.

	*n* (%)
Analysis set	138
Patients with adverse reactions	15 (10.87)
Thirst	6 (4.35)
Injection site pain	3 (2.17)
Dysphagia	2 (1.45)
Neck pain	2 (1.45)
Dry mouth	1 (0.72)
Malaise	1 (0.72)
Rash	1 (0.72)
Vision blurred	1 (0.72)

## 4 Discussion

This post-marketing surveillance is the first study to evaluate the real-world efficacy of botulinum toxin type B in Japanese CD patients who have received prior type A therapy. A total of 150 patients were registered at 17 Japanese study centers, and 138 of these were followed for 1 year. Both the TWSTRS total score and pain subscore improved from baseline to each evaluation time point, and this tendency for improvement was maintained during six injections. These data support and confirm the results of a previous double-blinded study in which 122 patients who had previously received botulinum toxin type A were treated with type B or placebo; in that analysis, a significant improvement was observed in TWSTRS total score and all subscores, as well as in other clinical rating scales, in the type B group [18]. The mean number of injections in the present study was 3.6 (range: 1–6), and more than half of the patients (57.25%) received four or more injections in the 1-year observation period. This indicates a favorable response to treatment over time in the majority of patients; the main reason patients discontinued treatment was that the drug was considered ineffective at the first injection, rather than diminished efficacy after subsequent administrations. In the present study, TWSTRS total score at Week 4 improved by 5.6 points. Recent studies of type A reported a TWSRTS total score improvement of 8.7–10.8 at Week 4 [[19], [20], [21]]. In this study, the maximum first injection dose was limited to 5000 units; Kaji et al. reported that 10,000 units of type B was associated with greater TWSTRS total score improvement at Week 4 (10.5) compared with 5000 units [15]. Thus, the lower maximum first dose used in the present study might have led a smaller change in total score. In addition, only patients with good response remained in the latter half of the study. We confirmed that the trend of score improvement was maintained even with repeated dosing in an incomplete longitudinal data analysis using the mixed-effects of model for repeated measures (data not shown). As such, type B is expected to be effective, with adequate pain control, in patients who respond to the first injection.

Subgroup analysis showed that type B had similar efficacy at both the lower maximum dose (≤5000 units) and higher maximum dose (>5000–10,000 units). These findings are in line with those from a prior dose-response study in Japan where both higher doses of type B (5000 and 10,000 units) and a lower dose of 2500 units were effective, compared with the placebo group, in reducing the total TWSTRS score at Week 4 after a single injection [15]. In the present study, the mean baseline total score for the lower and higher maximum dose groups was 38.2 and 43.5, respectively, indicating that a higher dose was more likely to be prescribed in patients with more severe baseline symptoms. It is of meaningful clinical relevance that type B efficacy was observed even at lower doses, to ensure optimal efficacy and safety based on patient symptoms, and also to minimize the risk of treatment failure potentially attributable to higher antigenicity at higher protein load [22]. Of note, patients who switch from botulinum toxin type A require careful tailoring of the type B dosage as equivalence cannot be assumed within and between the different serotypes [23]. While the presence of antigenic proteins against type B has been detected during treatment [24], more than half of the patients in the present study continued treatment until the fourth injection, and the remainder continued treatment until the sixth injection without experiencing severe AEs. The presence of botulinum toxin type B antibody may be a possible contributing factor to the diminished effects of treatment [25]. Although we did not evaluate the presence of these antibodies, we found that only 2.7% of patients discontinued due to diminished efficacy; this was comparable to the discontinuation rate with type A [21].

In the subgroup analysis of patients with or without type A-resistance, type B was shown to be similarly effective in both subpopulations at the 4-week evaluation after the first injection, and the tendency for improvement of symptoms including pain persisted in both groups until the sixth injection. In a previous multi-center double-blind randomized trial, 109 patients without type A resistance received either 5000 or 10,000 units of type B, or placebo. Patients who received the higher dose of type B showed a significant improvement in TWSTRS total score at 4, 8, and 12 weeks [26]. To the best of our knowledge, few data are available on the efficacy of type B in patients with type A-resistance compared with those without type A resistance. A previous study by Factor et al. reported that while the magnitude of response decreased with time, the objective efficacy was maintained until session 10 and the TWSTRS score did not decrease [27]. Our study findings support these results in a real-world large-scale setting, and are important for sequential therapy decision-making in CD patients.

In this study, among the patients who discontinued due to AEs, the main reason was thirst. It is known that botulinum toxin type B causes a higher frequency of dry mouth than type A, which might be explained by a serotype-specific variation in diffusion [11]. This characteristic of decreased salivation may suggest the potential use of type B for patients with parkinsonism presenting with sialorrhea. Further studies evaluating safety are, therefore, warranted.

A limitation of this study was the small sample size and nonavailability of sufficient efficiency evaluation data after the fourth injection, meaning that statistical significance of the change in TWSTRS score after the fifth and sixth injection was not demonstrated, particularly in the subgroup analysis. Another limitation was that, due to the nature of the observational study design, we observed patients over specific time points and not according to a specific number of doses; thus, the number of injections varied among patients. Moreover, the lack of clinical benefit may be attributable to various factors, including the presence of botulinum toxin type B antibody or technical issues, such as inadequate dosing, failure to accurately identify and inject the appropriate muscle, or difficulty targeting the intended muscle. However, most of the clinical investigators in this study had also participated in a clinical trial of onabotulinum toxin A, and it is likely that the quality of injection techniques was sound. Nonetheless, further studies with longer observational periods, conducted in larger patient populations, and evaluating additional factors that may influence treatment outcome, such as the presence of botulinum toxin type B antibody, are needed to confirm these results.

In conclusion, the results of this post-marketing study support the efficacy of type B in clinical settings for managing the symptoms of CD, including pain, even at low doses and independent of prior botulinum toxin type A resistance.

## Funding

This study was conducted and funded by Eisai Co., Ltd. Analysis and publication support for this study was funded by Eisai Co., Ltd. The study sponsor was involved in the conception and design of the study; data collection, analysis, and interpretation; and the writing and final approval of the manuscript.

## Declaration of Competing Interest

Ryuji Kaji commissioned the medical expert for this study from Eisai Co., Ltd., and received honoraria from Eisai Co., Ltd. Akira Endo, Michiko Sugawara, and Mika Ishii are employees of Eisai Co., Ltd.

## Appendix A Supplementary data

Supplementary material 1 Precautions for type B dosage and administration. Supplementary material 1
