# Supplementary material for: Efficacy of botulinum toxin type B (rimabotulinumtoxinB) in patients with cervical dystonia previously treated with botulinum toxin type A: A post-marketing observational study in Japan
Source: eNeurologicalSci. 2021 Oct 27;25:100374. doi: 10.1016/j.ensci.2021.100374 (PMC8627969; doi:10.1016/j.ensci.2021.100374)
Supplement: Supplementary material 1 — Precautions for type B dosage and administration. [file mmc1.docx]

**Supplementary Material**

**Precautions for type B dosage and administration**

(1) The potency (units) of type B is unique to this drug and differs from that of other botulinum toxin preparations, including botulinum toxin type A. The dosage therefore cannot be converted between preparations, meaning that the dosage of type B should be carefully considered when starting treatment.

(2) If the affected muscle is difficult to identify by palpation (for example, if the muscle location is deep), the target site should be carefully identified using electromyography.

(3) If sufficient efficacy is not observed, the dose and injection site should be re-evaluated prior to the next injection of type B.

(4) Following a reduction in target muscle tone after type B injection, increased tone in the adjacent muscles may be observed in some cases, leading to abnormal posture. It is therefore necessary to carefully identify the target tensor muscles for subsequent injections.

(5) If effective tone reduction is not observed after the first or second injection, discontinuation should be considered because efficacy may not be achieved, even with higher injection frequency or dosage.

(6) Type B should be injected with care given to the appropriate injection site and dosage for each muscle.

(7) Type B should not be used concomitantly with botulinum toxin type A. No efficacy and safety data are available for concomitant use of type B and botulinum toxin type A. If both agents are used concomitantly, paralysis of the neuromuscular junction may be enhanced, leading to serious adverse events such as dyspnea and dysphagia.

(8) When injecting type B after treatment with botulinum toxin type A, it is necessary to preserve the prescribed interval of botulinum toxin type A and to inject type B only after the effects of botulinum toxin type A have attenuated. Patients should be carefully observed for any adverse events. Note that the efficacy and safety of type B use within 3 months after botulinum toxin type A injection have not been established.

**Fig. S1** Flow chart of patient inclusion in the efficacy analysis set.

**Fig. S2** Mean (95% CI) change (decrease) in TWSTRS severity score

from baseline. The final observation was 4 weeks after the final injection.

*p < 0.05

CI, confidence interval; TWSTRS, Toronto Western Spasmodic Torticollis Rating Scale; w, weeks

**Fig. S3** Mean (95% CI) change (decrease) in TWSTRS dysfunction score

from baseline. The final observation was 4 weeks after the final injection.

*p < 0.05

CI, confidence interval; TWSTRS, Toronto Western Spasmodic Torticollis Rating Scale; w, weeks

**Table S1.** Injection site and recommended dose of type B.

| Dosing muscle | Recommended initial dose ^c^,  number of injection sites | Maximum dose ^d^ |
| --- | --- | --- |
| Sternocleidomastoid ^a^ | 625–1500 units divided into two or more injections | 4000 units |
| Scalenus | 500–1250 units | 2500 units |
| Trapezius | 750–2000 units divided into two or more injections | 4000 units |
| Levator scapulae ^b^ | 625–1250 units | 2500 units |
| Splenius capitis | 1000–2500 units divided into two or more injections | 5000 units |
| Semispinalis capitis | 500–1250 units | 2500 units |

^a^ Injection to the sternocleidomastoid on both sides should be avoided to reduce the risk of dysphagia.

^b^ When injecting into the levator scapulae, care should be taken to avoid increased dysphagia and respiratory infection.

^c^ Recommended dose of first injection in each muscle.

^d^ Recommended dose to each injection site is up to 1000 units, and the maximum recommended dose to each injection site is up to 2500 units
